# Multiple hormone deficiency syndrome: a novel topic in chronic heart failure

**DOI:** 10.4155/fsoa-2018-0041

**Published:** 2018-04-16

**Authors:** Andrea Salzano, Antonio Cittadini, Eduardo Bossone, Toru Suzuki, Liam M Heaney

**Affiliations:** 1 Department of Cardiovascular Sciences & NIHR Leicester Cardiovascular Biomedical Research Centre, University of Leicester, Glenfield Hospital, Leicester LE3 9QP, UK; 2 Department of Translational Medical Sciences, University Federico II of Naples, Naples, Italy; 3 Heart Department, Cardiology Division, “Cava de'Tirreni & Amalfi Coast” Hospital, University of Salerno, Salerno, Italy; 4 School of Sport, Exercise & Health Sciences, Loughborough University, Loughborough, LE11 3TU, UK

**Keywords:** biomarkers, heart failure, hormone replacement therapy, hormones, personalized medicine

Heart failure (HF) is described as a clinical syndrome characterized by typical symptoms (e.g., ankle swelling, fatigue or dyspnea) or signs (e.g., peripheral edema, pulmonary crackles or elevated jugular venous pressure), in which structural and/or functional cardiac abnormalities induce an impairment of cardiac output or an increase of intracardiac pressures at rest and/or during stress [1,2]. Importantly, due to different underlying etiologies, demographics, co-morbidities, and response to therapies, the main terminology used to describe HF is based on measurement of left ventricle ejection fraction (EF). Classically, patients with normal EF (typically considered as ≥50%) are said to have HF with preserved EF (HFpEF), with those with reduced EF (typically considered as <40%) termed as HF with reduced EF (HFrEF). In the latest European Society of Cardiology guidelines, cases where EF lies between 40 and 49%, previously considered as a ‘gray area’, are now defined as HF with mid-range EF (HFmEF) [1].

Although in recent years overall mortality from cardiovascular disease has been reduced by about two-thirds, HF represents an exception to this rule, maintaining high levels of mortality that are known to be higher than those of many cancers [3].

This trend is not surprising if we consider the nature of the classical neurohormonal model that provides the pathophysiological basis for the natural progression of HF, resulting in the main target for drugs currently used in HF management (β-blockers, ACE-I/ARBs/ARNI, and aldosterone receptor blockers). This model involves hyperactivity of neurohormonal pathways (the renin–angiotensin–aldosterone pathway or the adrenergic system) that, although offering a compensatory role in early stages of disease, becomes a factor responsible for the worsening and progression of HF by triggering a cascade of further and deleterious increases in neurohormonal action. Moreover, it is well known that this model is present despite pharmacological interventions, via activation of alternative pathways (e.g., myocardial chimases) [4].

For these reasons, researchers are looking for alternative models that could improve the understanding of the mechanisms underpinning HF progression. In particular, a growing body of evidence suggests that in addition to the increase of the pathways regulated by neurohormonal hyperactivity, the loss of equilibrium between the activation of these catabolic pathways and the impairment of anabolic hormonal axes depict the progression of the disease [5].

In this context, in 2009 Sacca published an elegant review that first described the concept that HF could be considered as a multiple hormone deficiency syndrome (MHDS) [5]. Each component of MHDS (e.g., GH/IGF-1 axes, thyroid hormones, androgens, and insulin resistance) is associated with impaired functional capacity and poor clinical outcome. Furthermore, prognosis has been shown to relate to the number of coexistent deficiencies [6]. This is not surprising considering the important relationship between hormones and the cardiovascular system [7–10].

Recently, initial investigations conducted by our group [11,12], demonstrated that not only the prevalence of hormonal deficiency is elevated in HF [13,14] and consequently related to poor cardiovascular performance and prognosis [13–15], but also that targeted hormone replacement therapy [16,17] leads to an improvement in cardiovascular performance and outcomes. These data provide strong evidence to suggest that the reversal of MHDS should be considered as an exciting and novel strategy in HF management [18,19].

Each deficiency is associated with reduced functional capacity and is a powerful and independent predictor of poor clinical outcome [5]. In particular, Arcopinto *et al*. demonstrated that in a population of around 200 stable chronic HF patients, less than a fifth presented with no signs of hormonal deficiency [13]. Moreover, in patients with two or more deficiencies cardiovascular performance was impaired, as demonstrated by a decreased ability for maximal oxygen uptake (measured by a cardiopulmonary exercise test) and elevated levels of circulating NT-pro-BNP (a well-known marker of severity and prognosis in HF). Furthermore, the presence and number of deficiencies were related with a poor prognosis for all-cause mortality.

These data were in line with those presented by Jankowska *et al*. [6], who demonstrated that multiple anabolic deficiencies occur frequently in HF (only 10% of the diseased population presented with no hormonal deficiency) and can predict long-term outcome. In particular, they found that reduced levels of multiple serum anabolic hormones (testosterone, DHEAS, and IGF-1) are strong markers of poor prognosis that are independent of conventional risk predictors. This reinforced the relationship between the number of hormonal deficiencies and the subsequently profound impact on the progression of HF and associated outcomes.

Interestingly, Arcopinto *et al*. [13] demonstrated that HF heavily impacted the age-related decline of anabolic hormones. Specifically, this decline was attenuated for DHEAS and IGF-1, with a paradoxical inversion observed for testosterone. This could suggest that the anabolic decline is age-independent and that the impact of MHDS could be more severe in young patients. This phenomenon could clearly have an impact on quality of life of patients diagnosed at an earlier age.

In 2016, a similar finding was demonstrated for the first time in HFpEF patients [14]. Despite in this group, fewer patients presenting with reduced levels of MHDS compared with an HFrEF population, a remarkable prevalence of hormonal deficiency was noted. In particular, approximately 45% of the study population displayed two or more signs of hormone deficiency. However, the fact that there is a higher level of anabolic drive in HFpEF could further support the notion that HFpEF should be considered as distinct from HFrEF. Also for HFpEF, anabolic deficiencies could provide additional utility for prognostic biomarker investigations, as well as identifying hormonal systems for targeted and personalized treatment strategies.

Considering the importance of the findings on MHDS and HF to date, the T.O.S.CA. Registry is an important trial to further understand the implications of hormonal treatments on HF severity and progression [20]. Results from this prospective multicenter observational outcome-oriented study, designed to evaluate the prevalence of MHDS and its impact on clinical outcomes in patients with chronic HF, are expected to provide important milestones surrounding this topic.

In conclusion, we recommend that screening for MHDS should be routinely performed in patients diagnosed with chronic HF, with a view to better characterize its impact on disease. This stance is taken due to the elevated prevalence of MHDS in HF and its impact on disease progression, coupled with preliminary data derived from small studies that show an improvement in cardiovascular performance and patient outcome following hormonal replacement therapy.
